# Human iPSC‐Derived Vascularised Lung Organoids for Modelling COPD and Pulmonary Hypertension

**DOI:** 10.1111/cpr.70273

**Published:** 2026-09-01

**Authors:** Simin Jiang, Liangliang Tian, Jingge Qu, Yi Hong, Dian Chen, Zihang Pan, Huanyu Long, Lanhe Chu, Weijing Kong, Qiyang Yao, Xiaojing Ma, Yun Zhao, Baihui Ma, Kai Wang, Yahong Chen

**Affiliations:** ^1^ Department of Pulmonary and Critical Care Medicine, State Key Laboratory of Natural and Biomimetic Drugs Peking University Third Hospital Beijing China; ^2^ Department of Physiology and Pathophysiology, School of Basic Medical Sciences, State Key Laboratory of Vascular Homeostasis and Remodeling, Beijing Advanced Center of Cellular Homeostasis and Aging‐Related Diseases, Clinical Stem Cell Research Center Peking University Third Hospital, Peking University Beijing China; ^3^ Department of Cardiac Surgery Peking University Third Hospital Beijing China; ^4^ State Key Laboratory of Cardiovascular Disease, Beijing Key Laboratory for Molecular Diagnostics of Cardiovascular Diseases, Diagnostic Laboratory Service Fuwai Hospital, National Center for Cardiovascular Diseases, Chinese Academy of Medical Sciences and Peking Union Medical College Beijing China

**Keywords:** blood vessel, chronic obstructive pulmonary disease, lung organoid, pulmonary hypertension

## Abstract

Vascularised lung organoids (vLOs) that faithfully mimic human lung tissue architecture and disease pathology are critical for advancing pulmonary research but remain challenging to generate. Here, we developed a robust self‐organisation protocol to produce vLOs with cellular heterogeneity and functional vasculature. The engineered blood vessels within vLOs exhibit lung‐specific characteristics. As proof of concept, we applied this platform to model chronic obstructive pulmonary disease (COPD) and pulmonary hypertension (PH). Using patient‐derived vLOs, we demonstrated that cigarette smoke extract (CSE) induces pathological features resembling clinical COPD, including epithelial disruption and inflammatory responses. Furthermore, vLOs generated from a PH patient recapitulated disease‐associated vascular remodelling, with RNA‐seq revealing dysregulated pathways in endothelial dysfunction. Notably, we identified sodium hydrosulfide (NaHS) as a potential therapeutic candidate, as it attenuated aberrant EndoMT in PH‐vLOs. In summary, our study establishes a physiologically relevant vLO system that enables modelling of cell‐type‐specific disease mechanisms and underscores the broad utility of this platform for mechanistic investigations and precision medicine approaches in respiratory disorders.

## Graphical abstract

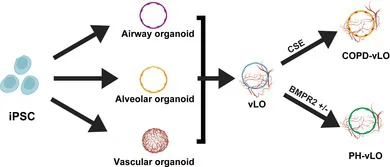

## 1 Introduction

The lung is a highly complex organ with multiple cell types, intricate structures and specialised functions. Human induced pluripotent stem cells (iPSCs)‐derived three‐dimensional (3D) lung organoids offer substantial potential for investigating human lung development and pathologies. Various models—including airway, alveolar and lung bud organoids—have been employed to recapitulate pulmonary diseases like infections, cystic fibrosis, idiopathic pulmonary fibrosis and chronic obstructive pulmonary disease (COPD) [1, 2, 3, 4]. These overcome barriers to accessing human lung tissue samples from non‐surgical patients. Meanwhile, lung organoids derived from CRISPR/Cas9‐engineered iPSCs (carry specific mutations alongside gene‐corrected counterparts) provide powerful tools for studying monogenic disorders [5], collectively providing unlimited resources for pulmonary disease modelling.

However, lung organoids lack a functional vasculature, which limits their utility in studying pulmonary vascular diseases and vascular injuries associated with other pulmonary disorders. A recent approach has achieved vascularised lung organoids through the co‐development of mesoderm and endoderm [6]. Here, we provide a straightforward method that co‐cultures vascular organoids with lung organoids to generate vascularised organoids, enabling the modelling of pulmonary diseases that affect both the lung epithelium and the vasculature.

Pulmonary hypertension (PH)—characterised by vascular remodelling, endothelial cell (EC) dysfunction and smooth muscle cell (SMC) proliferation [7]—has been modelled using PH patient‐derived iPSCs differentiated into ECs/SMCs [8, 9, 10]. Compared to monolayer‐differentiated cells, 3D vascularised lung organoids (vLOs) provide a superior platform for studying PH pathogenesis by recapitulating dynamic crosstalk among ECs, SMCs and lung epithelial cells—an integrated system whose dysregulation underlies PH development. Beyond primary vascular diseases, parenchymal disorders like COPD exhibit strong links to vascular pathology: 30%–70% of COPD patients develop comorbid PH, with pulmonary artery pressure directly correlating with disease severity [11]; furthermore, a distinct subgroup demonstrates disproportionately elevated pulmonary artery pressure relative to pulmonary dysfunction—termed the ‘pulmonary vascular phenotype’ [12]; and even those without overt PH exhibit pulmonary vascular remodelling, characterised by intimal‐medial thickening of pulmonary arteries and precapillary arteriole muscularisation [13]. By recapitulating vasculature in 3D organoid systems, vLOs thus emerge as superior models for investigating COPD pathogenesis and other parenchymal disorders coupling parenchymal and vascular disease processes. Collectively, vLOs overcome fundamental model limitations, enabling integrated study of vascular (PH) and parenchymal diseases with vascular comorbidities (COPD).

In this study, we established a robust self‐organisation protocol for generating vLOs that recapitulate the cellular heterogeneity and complex tissue architecture of native human lung tissue. Notably, the engineered vasculature within these vLOs demonstrates lung‐specific characteristics and significantly enhances organoid maturation. As proof‐of‐concept applications, we developed disease‐specific vLO models for COPD and PH. Using COPD patient‐derived vLOs, we demonstrated that cigarette smoke extract (CSE) treatment induces pathological features that closely mirror those observed in clinical COPD specimens. Furthermore, PH patient‐derived vLOs successfully modelled disease‐associated vascular remodelling and we identified sodium hydrosulfide (NaHS) as a potential therapeutic agent capable of attenuating these pathological changes.

## 2 Method

### 2.1 Generation of iPSCs Line

The iPS‐18 CD31‐tdTomato cell line was generated as described previously [14]. COPD patient‐derived iPSCs were established from a 60‐year‐old male with COPD (30 pack‐year smoking history) after obtaining written informed consent, with ethical approval granted by the Medical Science Research Ethics Committee of Peking University Third Hospital (M2021042). PH patient‐derived iPSCs were generated from an adult female carrying a pathogenic *BMPR2* mutation (c.631 C>T) at Fuwai Hospital's cardiovascular department, with patient consent and protocol approval by the Chinese Academy of Medical Sciences ethics committee (2017–877). The isogenic control PH iPSC line was created using CRISPR/Cas9 technology as detailed in prior publications [15, 16]. The primers and key reagents used in this study are listed in Tables S1 and S2.

### 2.2 Animals

NOD‐SCID interleukin‐2 receptor subunit gamma knockout (NTG) mice, a strain with immune deficiency, were purchased from SPF (Beijing) Biotechnology Co. Ltd. All mice were housed in the Specific Pathogen‐Free (SPF) Laboratory Animal Facility of Peking University Third Hospital under standardised conditions: temperature maintained at 25°C, with a 12‐h light/12‐h dark cycle. Food and water were provided ad libitum. Twelve‐week‐old mice (weight: 20–25 g) were used for transplantation experiments. The experimental protocol was approved by the Medical Science Research Ethics Committee of Peking University Third Hospital (Approval No.: A2022132).

### 2.3 Generation of Isogenic BMPR2‐Corrected iPSC Line

We used the CRISPR‐Cas9 system to generate an isogenic BMPR2‐corrected iPSC line [15]. Briefly, PH iPSCs were dissociated with EDTA for 7 min at 37°C and neutralised in mTeSR medium containing 5 μM Y‐27632 dihydrochloride at a density of 10^7^ cells/mL. For electroporation, 10^6^ cells were resuspended in 100 μL of the same medium and mixed with 2 μg donor plasmid and 1 μg Cas9 plasmid in a 1.5 mL tube. Nucleofection was performed using the Lonza Nucleofector 2B with program B‐016. After electroporation, cells were seeded into one well of a Matrigel‐coated six‐well plate containing 2 mL mTeSR with 5 μM Y‐27632. The medium was refreshed 24 h post‐editing and Y‐27632 was removed after another 24 h. Puromycin (0.5 μg/mL) was added for 2 days to select and enrich successfully edited cells. Subsequently, approximately 3000 cells were plated onto a 10‐cm dish for colony formation and picking. Correct insertion and off‐target effects were verified by junction PCR and off‐target site PCR.

### 2.4 Generation of Lung Progenitor Cells

iPSCs were dissociated with 0.5 mM EDTA and replated at 80%–90% confluence on day 0. For definitive endoderm differentiation, cells were cultured in MCDB131 basal medium consisting of 11.6 g/L MCDB131 (Sigma‐Aldrich, M8537), 0.44 g/L D‐glucose, 2.68 g/L NaHCO_3_, 5 g/L Bovine serum albumin (Proliant, 68700), 80 μg/mL L‐ascorbic acid (Sigma‐Aldrich, A8960), 10 mL GlutaMAX (Thermo Fisher Scientific, 35050061) and 1 mL penicillin–streptomycin (Thermo Fisher Scientific, 15‐140‐122) in 1000 mL ddH_2_O. On day 1, cells were cultured in MCDB131 basal medium supplemented with 100 ng/mL activin A (Sino Biological, 10429‐HNAH) and 3 μM CHIR99021 (Topscience, T2310), followed by 100 ng/mL activin A alone on days 2–3. For subsequent differentiation stages, the DMEM/F12 basal medium was prepared with 500 mL DMEM/F12 (Gibco, C11330500BT) supplemented with 0.25 g BSA (Proliant, 68700), 50 μg/mL L‐ascorbic acid (Sigma‐Aldrich, A8960), 5 mL GlutaMAX, 5 mL N2 (Yeasen, 60706ES08), 10 mL B27 (Yeasen, 60703ES10), 20 μL β‐mercaptoethanol (Sigma‐Aldrich, M3148) and 1 mL penicillin–streptomycin. Definitive endoderm cells were passaged at a 1:3 ratio and then differentiated into anterior foregut endoderm in DMEM/F12 basal medium containing 2 μM dorsomorphin (DSM) (TargetMol, T6146) and 10 μM SB431542 (Topscience, T1726) for 3 days. Anterior foregut endoderm was then further differentiated into lung progenitor cells by using DMEM/F12 basal medium supplemented with 3 μM CHIR99021, 10 ng/mL BMP4 (Novoprotein, CR93) and 50 nM retinoic acid (Sigma‐Aldrich, R2625) for 9 days.

### 2.5 Isolation of Lung Progenitors

Lung progenitor cells were isolated using MojoSort Human anti‐PE Nanobeads per manufacturer's protocol. Single‐cell suspensions were prepared by dissociation and 70 μm filtration. Cells were then: (1) blocked with Human TruStain FcX, (2) incubated with anti‐CPM primary antibody (4°C, 10 min), (3) labelled with PE‐conjugated secondary antibody (4°C, 10 min) and (4) mixed with anti‐PE Nanobeads (4°C, 15 min). CPM + cells were finally isolated by magnetic separation.

### 2.6 Generation of Lung Organoids

Lung progenitor cells were embedded in growth factor‐reduced Matrigel (Corning, 354,230) (400 cells/μL) and cultured for 1 week by using DMEM/F12 basal medium supplemented with CHIR99021 (3 μM) and KGF (10 ng/mL, Sino Biological, 10210‐H07E). Subsequently, alveolar organoids were induced by adding CHIR99021 (3 μM), KGF (10 ng/mL) and DIC cocktail [50 nM dexamethasone (TargetMol, T1076) + 100 μM IBMX (TargetMol, T1713) + 100 μM cAMP (8‐Bromo‐cAMP, TargetMol, T6747)] alongside CHIR99021 and KGF for 2 weeks. Alternatively, airway organoids were generated using DMEM/F12 basal medium supplemented with FGF2 (250 ng/mL, Sino Biological, 10014‐HNAE), FGF10 (100 ng/mL, Sino Biological, 10573‐HNAE), DAPT (2 μM, TargetMol, T6202) and the DIC cocktail.

### 2.7 Generation of Vascular Organoids

To generate vascular organoids, iPSCs were dissociated and replated at 30% confluence. We prepare LaSR basal medium by adding GlutaMAX (1%) and L‐ascorbic acid (50 μg/mL) in Advanced DMEM/F12 (Thermo Fisher Scientific, 12634028). Mesoderm induction was achieved by daily treatment with LaSR basal medium supplemented with CHIR99021 (6 μM) for 3 days. At day 3, mesoderm cells were dissociated and resuspended (15,000 cells/well in 4 mL medium) in non‐treated six‐well plates with LaSR basal medium supplemented with vascular differentiation factors: FGF2 (50 ng/mL, Sino Biological, 10014‐HNAE), VEGF (50 ng/mL, Sino Biological, 11,066‐HNAH), L690330 (10 μM, R&D systems, 0681), SB431542 (10 μM) and EGF (10 ng/mL, Sino Biological, 10,605‐HNAE). Plates were then agitated on an orbital shaker (130 rpm) for 3 days to promote vascular organoid formation.

### 2.8 Generation of vLOs

At day 35, alveolar and airway organoids were harvested from Matrigel using cell recovery solution (Corning Biocoat, 354253) on ice for 10 min. The organoids were then combined with vascular organoids in a 2:2:1 ratio and embedded in a Matrigel/Collagen I (1:1) mixture (Collagen I: Sigma‐Aldrich, 5074). Cultures were maintained in DMEM/F12 basal medium supplemented with VEGF (50 ng/mL), FGF2 (250 ng/mL), FGF10 (100 ng/mL), KGF (10 ng/mL) and the DIC cocktail. Vascular networks typically formed within 7 days.

### 2.9 Measurement of the Area of the Cultivation Matrices of vLOs

To assess vascular contractility, vLOs embedded in collagen I/Matrigel mixture were gently detached from the culture dish walls and bottom using a curved‐tip metallic spatula to allow free‐floating contraction. Culture media with or without CSE were renewed every 48 h. Gel contraction was quantified at day 1 and day 7 by measuring the major and minor axes of each gel using a Stereo Microscope (Chongqing Optec Instrument, SZ680). The gel area was calculated from the averaged diameters and expressed as a percentage of the initial area.

### 2.10 Isolate Single‐Cell Suspension From vLOs

Vascularised organoids were enzymatically digested in PBS containing 3 U/mL dispase (Stemcell, 07923) plus 2 mg/mL each of Collagenase I (Gibco, 17101015) and Collagenase II (Gibco, 17100017) at 37°C for 40 min with constant rotation. After enzymatic treatment, the dissociated cells were washed with PBS and passed through 40 μm cell strainers to generate a single‐cell suspension.

### 2.11 Transplantation of Human Lung and Vascular Organoids

On day 35 of differentiation, airway and alveolar organoids were harvested from Matrigel and combined with vascular organoids at a 2:2:1 ratio. The organoid mixture was resuspended in ice‐cold PBS at a concentration of 2 × 10^6^ per 100 μL. For transplantation, 50 μL aliquots of the suspension were injected beneath the renal capsule of recipient mice. Given the established oestrogen‐dependent gender disparity in PH incidence, all organoids were derived from female donors (either PH patients or isogenic controls) and transplanted exclusively into female NSG mice to maintain gender‐matched experimental conditions.

### 2.12 Blood Perfusion of Transplanted Vessels Detection by Two‐Photon In Vivo Imaging

CD31‐tdTomato labelled vascular organoids were co‐transplanted with wild‐type airway/alveolar organoids under the renal capsule. Two weeks post‐transplantation, anaesthetised mice received tail vein injections of Evans blue (2 mg/mouse). For imaging, kidneys were surgically exposed and placed in a custom light‐stimulation chamber. Transplanted organoids were gently aspirated onto 5‐mm coverslips to create an imaging plane, then visualised using a Leica‐controlled two‐photon microscope.

### 2.13 Quantitative Real‐Time PCR Analysis

Total RNA was isolated using the FastPure Cell/Tissue Total RNA Isolation Kit V2 (Vazyme). cDNA synthesis and qPCR were performed with HiScript III RT SuperMix Kit (Vazyme, R323‐01). All reactions were run in technical duplicates/triplicates, with Ct values averaged for analysis. Gene expression was normalised to GAPDH and calculated as fold change using the 2^−ΔΔCT^ method. Experiments included ≥ 3 biological replicates.

### 2.14 Flow Cytometry Analysis

Single‐cell suspensions were prepared by digestion and 40 μm filtration. For intracellular staining (SMA), cells were fixed (2% PFA, 37°C, 10 min), permeabilised (0.2% Triton‐X, 10 min), washed (300 g × 3 min) and blocked in FACS buffer (BD). Cells were stained with primary antibodies (fluorophore‐conjugated or unconjugated) for 20 min on ice (light‐protected). Unconjugated primaries required fluorescent secondary antibodies. Surface markers (MUC5AC/CD31/PDGFRβ) were stained without fixation/permeabilisation. Samples were analysed on BD FACS Aria II or Beckman CytoFlex instruments, with data processed using FlowJo (v10.6.2).

### 2.15 Immunofluorescence Staining

Paraffin‐embedded tissue or organoid samples were sections (6 μm), deparaffinised (xylene I/II, 5 min each) and rehydrated (graded ethanol, 5 min each). Antigen retrieval was performed in preheated EDTA buffer (pH 8.0, Solarbio, C1033) (90°C–95°C, 30 min). After cooling, sections were blocked (5% horse serum, 30 min), incubated with primary antibodies (e.g., Rabbit anti‐NKX2.1, abcam, ab76013) (4°C, overnight) and stained with fluorescent secondaries (e.g., Donkey anti‐rabbit IgG, Alexa Fluor 568, Thermo Fisher Scientific, A10042) (1 h, RT). Nuclei were stained by Fluoroshield Mounting Medium with DAPI (Abcam, ab104139).

### 2.16 3D Organoid Clearing for 3D Fluorescence Imaging

Organoids were fixed with 4% paraformaldehyde and dehydrated through a graded ethanol series (50% in PBS, 80% in ddH_2_O, 100% ethanol; 10 min each with gentle shaking). They were then washed sequentially in 20% DMSO in 80% ethanol, 80% ethanol in ddH_2_O, 50% ethanol in PBS, 100% PBS and finally in PBS with 2% Triton X‐100 for 1 h. Organoids were blocked at room temperature for 1 h, incubated with primary antibodies diluted in blocking buffer at 4°C overnight, washed 10 times in wash buffer and then incubated with fluorescence‐conjugated secondary antibodies for 1 h. After another 10 washes, nuclei were stained with DAPI (Thermo Fisher Scientific, Cat# D1306, USA). Finally, organoids were dehydrated through a graded ethanol series and embedded in CytoVista Tissue Clearing Reagent (Thermo Fisher Scientific, v11326). The details of the protocol referred to the CytoVista Tissue Clearing Reagent manual.

### 2.17 Western Blot Analysis

Protein was isolated from cultured cells using RIPA Buffer with protease inhibitors (30 min on ice) and concentration was measured via BCA kit (Beyotime, P0012). Twenty micrograms of lysate was loaded onto 10% SDS‐PAGE, then transferred to PVDF membranes. Membranes were blocked with 5% BSA in TBST (0.1% Tween‐20, Solarbio, T8220) for 1 h at room temperature, incubated with primary antibodies at 4°C overnight, washed 3× with TBST (10 min each, gentle shaking), then incubated with horseradish peroxidase‐conjugated secondary antibody for 1 h at room temperature. After three more washes, membranes were treated with ECL Reagent (Beyotime, P0018S); targeted bands were visualised via enhanced chemiluminescence system and signal density quantified using Image J.

### 2.18 Haematoxylin and Eosin (HE) Staining and Masson Staining

For HE or Masson staining, sections were first deparaffinised and rehydrated. For HE staining: stain with Harris haematoxylin (5–8 min, RT), rinse with tap water, differentiate in 1% hydrochloric acid/70% ethanol (3–5 s) then rinse, immerse in tap water (10–15 min) for nuclear bluing, stain with eosin Y (2–3 min) and rinse with distilled water. For Masson staining: after deparaffinisation/rehydration, stain with Weigert's iron haematoxylin (Masson kit, 5 min), rinse, differentiate in 1% hydrochloric acid alcohol (few seconds), rinse and wash with running water until nuclei blue; stain with acid fuchsin (Masson kit, 5–10 min), rinse, incubate with phosphomolybdic acid (Masson kit, 3–5 min) without prior washing, counterstain with aniline blue (Masson kit, 5 min) and treat with 1% acetic acid (1 min). Both concluded with dehydration in 95% ethanol (2 × 3 min for HE, 2 × 5 min for Masson), 100%/anhydrous ethanol (2 × 3 min for HE, 2 × 5 min for Masson), xylene (2 × 5 min) and mounting with neutral balsam.

### 2.19 Cell Viability Assay

Cell viability was assessed using a CCK‐8 kit (Yesean, 40203ES76). Induced EC and mural cells (5000/well) were seeded in 96‐well plates, cultured for 24 h to adhere, then treated with respective concentrations of NaHS for 24 h. The medium was replaced with 90 μL DMEM/F12 + 10 μL CCK‐8 solution; after 2 h incubation at 37°C, absorbance at 450 nm was measured with a microplate reader.

For organoid viability, vLOs were plated in 12‐well plates, treated with respective concentrations of CSE for 2 weeks, then incubated with 900 μL DMEM/F12 + 100 μL CCK‐8 solution at 37°C for 2 h. One hundred microlitres of the medium was transferred to 96‐well plates and absorbance at 450 nm was detected using a microplate reader.

### 2.20 Preparation of CSE

CSE was prepared by bubbling smoke from research‐grade cigarettes (3R4F; University of Kentucky) into 100 mL DMEM/F12, then filtering through a 0.22 μM filter. CSE concentration was standardised as 100% when its absorbance at 340 nm exceeded blank medium by 2.0. The medium was aliquoted into 1.5 mL EP tubes, stored at −80°C and unused CSE was discarded after each experiment to avoid freeze–thaw cycles.

### 2.21 PH‐Patient‐Derived vLOs Transplanted in Mice Model

After the vLOs had been transplanted in the mouse kidney capsule for 1 week, NaHS was administered intraperitoneally at a dose of 25 μmol/kg/day for an additional 3 weeks. Following the treatment, medial thickness was assessed in small pulmonary arteries (external diameter < 100 μm) within the transplanted tissue sections. Vessels were identified based on morphology and the presence of a defined endothelial layer (hCD31^+^) and smooth muscle layer (SMA^+^). Medial thickness was calculated as the ratio of the medial area to the external diameter using ImageJ software. Only cross‐sectional vessels with clearly defined lumens were selected for analysis.

### 2.22 ScRNA‐seq Data Processing and Integration

#### 2.22.1 Data Acquisition

Single‐cell RNA sequencing (scRNA‐seq) datasets of 51 COPD and 51 healthy control lung tissue samples (GSE173896, GSE136831, GSE167295, GSE196638, GSE171541, GSE196341 and GSE168191) were retrieved from the Gene Expression Omnibus (GEO) database (http://www.ncbi.nlm.nih.gov/geo/) [17, 18, 19, 20, 21, 22].

#### 2.22.2 Data Processing and Quality Control

Seven datasets containing scRNA‐seq data from 102 lung tissue samples and vLOs were analysed using the Seurat package in R 4.0.5. The initial quality control (QC) process involved several steps to assess and clean the data. Mitochondrial gene proportions were calculated for each cell to evaluate its health and QC metrics were visualised to identify potential issues. Additionally, the correlation between gene count, mitochondrial gene proportion and RNA molecule count was analysed to detect any technical or biological variations. These steps helped to ensure that only high‐quality data would proceed to the next stages of analysis.

#### 2.22.3 Cell Filtering

Following the QC process, cells were filtered based on predefined criteria to retain only high‐quality cells for subsequent analysis. The number of cells before filtering was recorded to assess the data scale. The filtering criteria included excluding cells with a mitochondrial gene proportion greater than 10%, ensuring the health of the cells and retaining only those with more than 500 expressed genes to guarantee sufficient gene expression data. Additionally, cells with RNA molecule counts between 1000 and 20,000 were included to avoid cells with either insufficient or excessive RNA content. The number of cells after filtering was also documented.

#### 2.22.4 Data Integration and Normalisation

The next step involved integrating the seven datasets into a single Seurat object using the merge function. To eliminate variations in sequencing depth across cells, the data were normalised using the NormaliseData function with the LogNormalise method, setting the scaling factor to 10,000. To focus on biologically relevant genes, variable features were selected from the normalised data using the FindVariableFeatures function with a variance‐stabilising transformation (vst) method. The top 2000 most variable genes were retained for further downstream analysis. Additionally, data were scaled using the ScaleData function, with mitochondrial gene proportions regressed out to minimise their impact on the data. PCA was then applied to the selected variable genes using the RunPCA function, reducing the dimensionality of the dataset.

#### 2.22.5 Batch Effect Correction and Dimensionality Reduction

To account for potential batch effects arising from differences between datasets, the RunHarmony function was applied to the COPD and healthy control lung tissue data, with the dataset source as the grouping factor. For the vLOs, the Anchor method was used to correct for batch effects. Specifically, the FindIntegrationAnchors function was applied to identify anchors between datasets. After batch effect correction, cell similarity was calculated using the K‐Nearest Neighbours (KNN) clustering algorithm to determine the optimal number of clusters. The FindNeighbors function was applied based on the batch‐corrected PCA results. Cell clustering was then performed using the FindClusters function, with a resolution parameter set to 0.4 to define the final clusters. Finally, dimensionality reduction and visualisation were carried out using the UMAP method with the RunUMAP function, based on the corrected PCA results and selected principal components, enabling clear visualisation of cell clusters in the lower‐dimensional space.

#### 2.22.6 Pseudotime Analysis

For vLOs exposed to CSE and non‐CSE controls, we performed pseudotime trajectory analysis using Monocle3 to investigate epithelial cell state transitions. Cells were ordered along a developmental trajectory based on gene expression profiles, with pseudotime reflecting their progression. Epithelial cells were classified into AT1/AT2 cells, ciliated cells, basal cells, goblet cells and undefined epithelial populations. The analysis revealed trajectories of CSE‐treated and control groups, highlighting shifts in epithelial cell states.

#### 2.22.7 Differential Expression Analysis

For vLOs exposed to CSE and non‐CSE controls, endothelial and epithelial cells were isolated and differential expression analysis was performed separately for endothelial and epithelial cells between the two groups. Similarly, for batch‐corrected lung tissues from COPD patients and healthy controls, endothelial and epithelial cells were extracted and differential expression analysis was conducted separately for each cell type to compare gene expression differences between the two groups.

#### 2.22.8 Enrichment Analysis

For endothelial and epithelial differential genes in vLOs exposed to CSE and non‐CSE controls, as well as for batch‐corrected lung tissues from COPD patients and healthy controls. Gene Set Enrichment Analysis (GSEA) were performed to uncover significantly enriched pathways related to CSE exposure and COPD pathology, providing insights into the molecular mechanisms driving these conditions.

## 3 Statistical Analysis

Statistical analyses were conducted using GraphPad Prism 9 (GraphPad Software, USA). Data are presented as mean ± standard error (SE). For multiple comparisons within groups, we performed one‐way analysis of variance (ANOVA) followed by either Tukey's post hoc test (for all pairwise comparisons) or Dunnett's test (for comparisons against a single control group), as appropriate. Between‐group differences were assessed using unpaired Student's *t*‐tests, while within‐group comparisons were evaluated using paired *t*‐tests. A *p*‐value < 0.05 was considered statistically significant.

## 4 Results

### 4.1 Generation and Characterisation of Vascularised Lung Organoid (vLO) With Lung Specific Endothelial Cells

**FIGURE 1:**
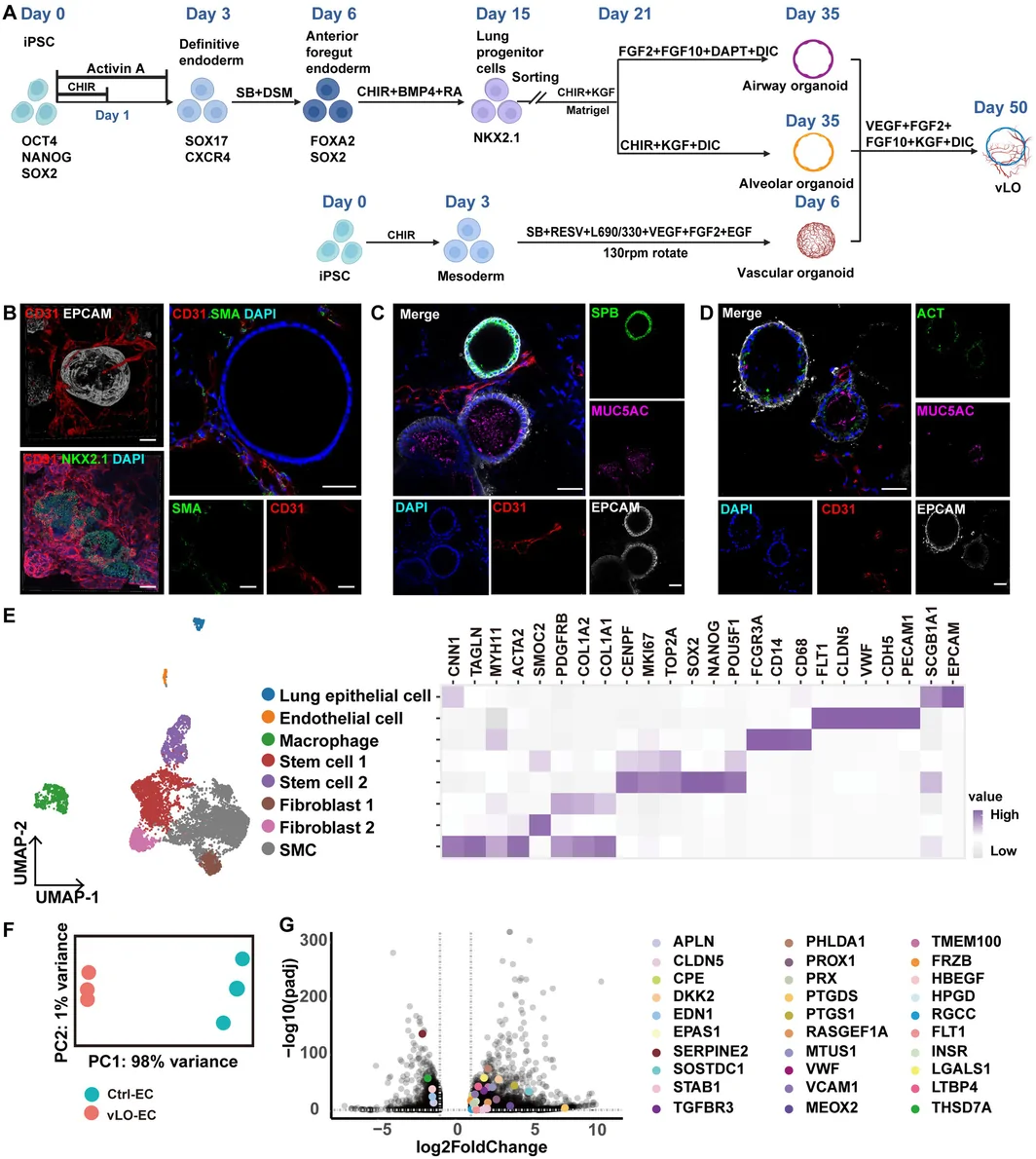
Generation and characterisation of vascularised lung progenitor organoids (vLOs). (A) Schematic diagram illustrating the differentiation protocol from induced pluripotent stem cells to vascularised lung organoid. (B) Structural characterisation of vLOs. 3D immunofluorescence staining demonstrates lung organoids (EPCAM^+^/NKX2.1^+^) embedded within a vascular network (CD31^+^). Cross‐sectional analysis reveals vascular lumen formation by endothelial cells (CD31^+^) and smooth muscle cells (SMA^+^) adjacent to lung epithelium. Scale bar: 50 μm. (C, D) Immunophenotypic profiling of vLO components: (C) Coexistence of alveolar epithelial cells (AT2; SPB^+^), airway goblet cells (MUC5AC^+^) and endothelial cells (EC; CD31^+^). (D) Endothelial cells (CD31^+^) with ciliated cells (ACT^+^) and goblet cells (MUC5AC^+^). CHIR: CHIR99021; CPM, carboxypeptidase M; DIC, dexamethasone + IBMX + cAMP; DSM, dorsomorphin; iPSCs, induced pluripotent stem cells; RA, retinoic acid; RESV, resveratrol; SB, SB431542. (E) Single‐cell RNA‐seq analysis of vLOs. Left: UMAP visualisation of cellular heterogeneity. Right: Heatmap of cluster‐defining marker genes. (F) Principal component analysis (PCA) of bulk RNA‐seq transcriptomes from vLO‐sorted ECs (vLO‐EC) versus control ECs cultured alone (Ctrl‐EC). (G) Volcano plot identifying significantly altered lung‐specific EC genes between vLO‐EC and Ctrl‐EC.

To generate vLOs containing the major spectrum of pulmonary epithelial cells, we co‐cultured airway, alveolar and vascular organoids differentiated from iPSCs. We established airway and alveolar organoid differentiation protocols by integrating and refining methodologies from published literature [23, 24, 25]. Detailed procedures are described in our prior work [15]. Briefly, iPSCs were differentiated into lung progenitor cells through sequential induction of definitive endoderm and anterior foregut endoderm, followed by magnetic bead sorting to purify Carboxypeptidase M^+^ (CPM^+^) cells (Figure 1A) [26]. Purified lung progenitor cells were embedded in Matrigel and expanded for 7 days. For alveolar organoid generation, cells were then treated for an additional 2 weeks with CHIR99021 and KGF, supplemented with the DIC cocktail (i.e., dexamethasone, IBMX and cAMP). For airway organoid induction, CHIR and KGF were withdrawn and the culture medium was supplemented with an FGF2/FGF10/DAPT cocktail alongside the DIC cocktail.

Given the prominence of vascular remodelling in small pulmonary arteries in pulmonary diseases [27], we preferentially differentiated iPSCs into artery‐like vascular organoids using the established protocol [14]. Briefly, iPSCs were differentiated into mesoderm via 3‐day treatment with CHIR99021, followed by exposure to an FGF2/VEGF/L690330/SB431542/EGF cocktail to induce arterial organoid formation.

On day 35, after successful generation of all organoid types, lung organoids were released from Matrigel. Airway organoids, alveolar organoids and vascular organoids were then combined at a 2:2:1 ratio and embedded in a 1:1 mixture of Matrigel and collagen I gel. The three‐organoid composite was cultured in medium supplemented with FGF10, FGF2, KGF, VEGF and DIC. Within 7 days, a fully interconnected vascular network developed throughout the embedded lung organoids, which could be maintained for at least 2 weeks. The overall strategy, along with phase‐contrast microscopy images depicting each stage of vLO generation, is shown in (Figure 1A and Figure S1A).

Three‐dimensional immunofluorescence imaging confirmed that lung organoids (EPCAM^+^, NKX2.1^+^) were integrated within this vasculature (Figure 1B, Movie S1). Cross‐sectional images further demonstrated the assembly of ECs (CD31^+^) and smooth muscle cells (SMCs, SMA^+^) forming vascular lumens adjacent to the lung organoids (Figure 1B). The resulting vLOs contained both alveolar (alveolar Type 2 cells [AT2], SPB^+^) and airway epithelial cells (goblet cells, MUC5AC^+^) (Figure 1C). Additionally, ciliated cells (ACT^+^), basal cells (KRT5^+^) and club cells (SCGB3A2^+^) were identified within the vLOs (Figure 1D, Figure S1B,C).

Having established structurally complete vLOs, we next examined whether the presence of vasculature confers lung‐specific endothelial identity and promotes epithelial maturation. Single‐cell RNA sequencing (scRNA‐seq) analysis on day 50 revealed a diverse cellular composition within the vLOs, including lung epithelial cells, ECs, SMCs, fibroblasts, macrophages and stem cells within the vLOs (Figure 1E). Notably, both epithelial and mesenchymal cell populations exhibited lung‐specific characteristics. The lung possesses two circulatory systems: the pulmonary circulation for gas exchange and the bronchial circulation for systemic blood supply. Within the pulmonary capillary network, lung‐specific aerocyte capillary (aCap) cells mediate gas exchange, whereas general capillary (gCap) cells serve as capillary stem cells [28]. Dot plot analysis of marker gene expression revealed that ECs within the vLOs expressed genes associated with bronchial ECs (PLVAP, HSPG2, HBEGF), gCap (PTPRB, EDN1, PLVAP) and aCap cells (S100A4, EMCN, HPGD) (Figure S1D) [28, 29]. Mesenchymal cells expressed transcription factors TBX3, TBX4 and TBX5, all of which have been implicated in the regulation of pulmonary endoderm specification (Figure S1E) [30, 31].

To further evaluate the impact of the lung microenvironment on endothelial identity, we performed bulk RNA sequencing to compare iPSC‐derived ECs cultured with versus without lung organoids (Figure 1F). ECs co‐cultured with lung organoids showed significantly higher expression of markers specific to lung ECs (Figure 1G) [6, 32]. This indicates that the lung microenvironment within vLOs actively reinforces organ‐specific endothelial identity.

### 4.2 Transplantation of vLO Forms Functional Vascular Perfusion

**FIGURE 2:**
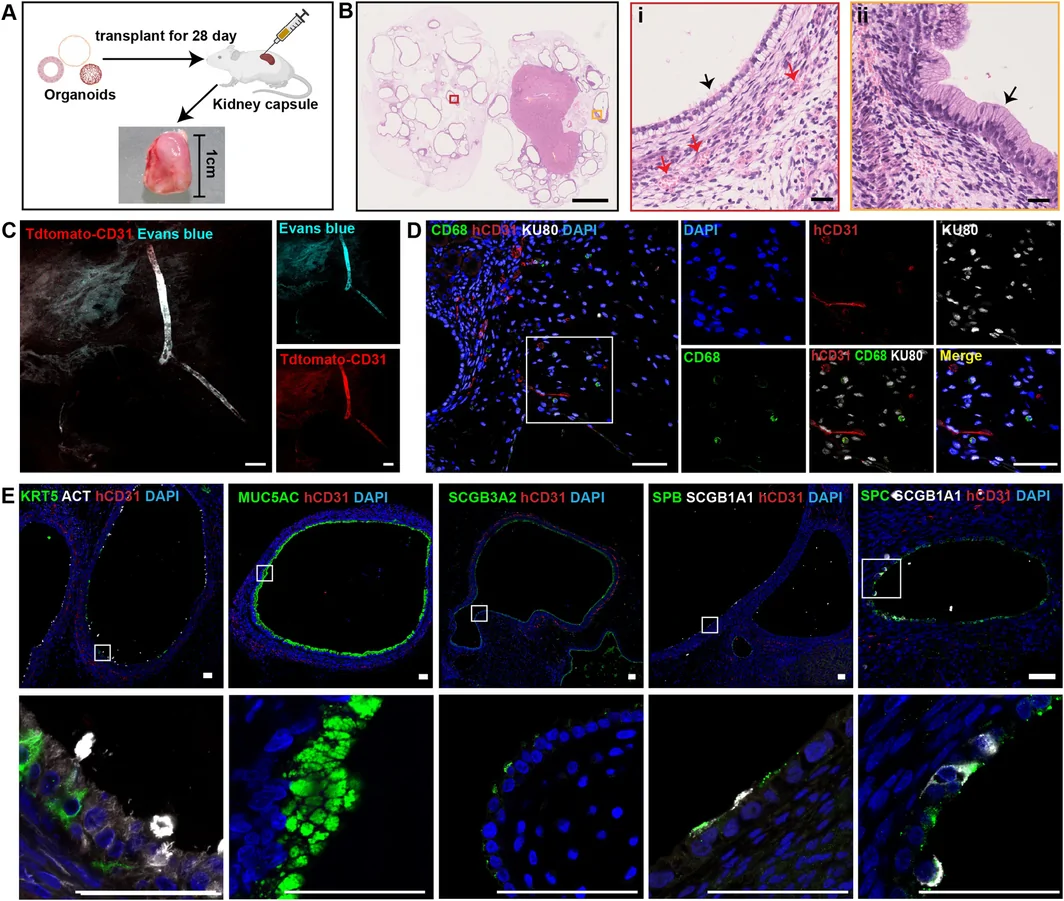
Functional vascular perfusion and engraftment of transplanted vLOs. (A) Schematic of the transplantation procedure showing vLO engraftment beneath the renal capsule and subsequent tissue harvest. (B) Haematoxylin and eosin (H&E) staining reveals perfused microvasculature adjacent to epithelial structures. (i) Ciliated epithelium (black arrows); vascular lumina containing erythrocytes (red arrows). (ii) Columnar airway epithelium (black arrows); Scale bar, 100 μm. (C) In vivo perfusion is confirmed by Evans Blue dye (administered intravenously) circulating through CD31‐tdTomato^+^ human vasculature. Scale bar, 50 μm. (D) Immunohistochemical characterisation of engrafted vLOs demonstrates the presence of human vascular (hCD31^+^) and macrophages (CD68^+^, KU80^+^). Scale bar, 50 μm. (E) Immunohistochemical characterisation of engrafted vLOs identifies: Human endothelial cells (hCD31^+^), basal cells (KRT5^+^), ciliated cells (ACT^+^), goblet cells (MUC5AC^+^), club cells (SCGB1A1^+^/SCGB3A2^+^), AT2 cells (SPB^+^, SPC^+^). Scale bar, 50 μm.

To establish a physiologically relevant in vitro model of human vascularised lung tissue, we transplanted a mixture of airway, alveolar and vascular organoids (in a 2:2:1 ratio) beneath the renal capsule of immunodeficient mice (Figure 2A). Kidneys were harvested 28 days post‐transplantation for histological analysis. Haematoxylin and eosin (H&E) staining revealed well‐integrated engrafted tissue structures (Figure 2B). At higher magnification, the grafts exhibited pseudostratified ciliated columnar epithelium lining luminal spaces, with adjacent blood vessels containing erythrocytes situated next to the epithelial layers (Figure 2B, i). Goblet cells were also clearly identifiable within the bronchial columnar epithelium (Figure 2B, ii).

To visualise the transplanted vasculature, we utilised CD31‐tdTomato‐labelled iPSC‐derived vascular organoids. Two‐photon imaging the presence of interconnected vascular networks and systemic administration of Evans Blue dye via tail vein injection demonstrated effective intravascular perfusion within these vessels (Figure 2C). The presence of human macrophages (CD68^+^, KU80^+^) was also observed within the transplanted tissue (Figure 2D).

The vascularised lung tissue contained a broad spectrum of lung epithelial cell types, including basal cells (KRT5^+^), ciliate cells (ACT^+^), goblet cells (MUC5AC^+^), club cells (SCGB3A2^+^ or SCGB1A1^+^), AT2 (SPB^+^), along with human vascular networks (hCD31^+^). The hCD31^+^ vasculature formed beneath the epithelial layer, exhibiting morphological characteristics resembling native human lung tissue (Figure 2E).

### 4.3 CSE Treated vLOs Recapitulate Pathological Features of COPD

**FIGURE 3:**
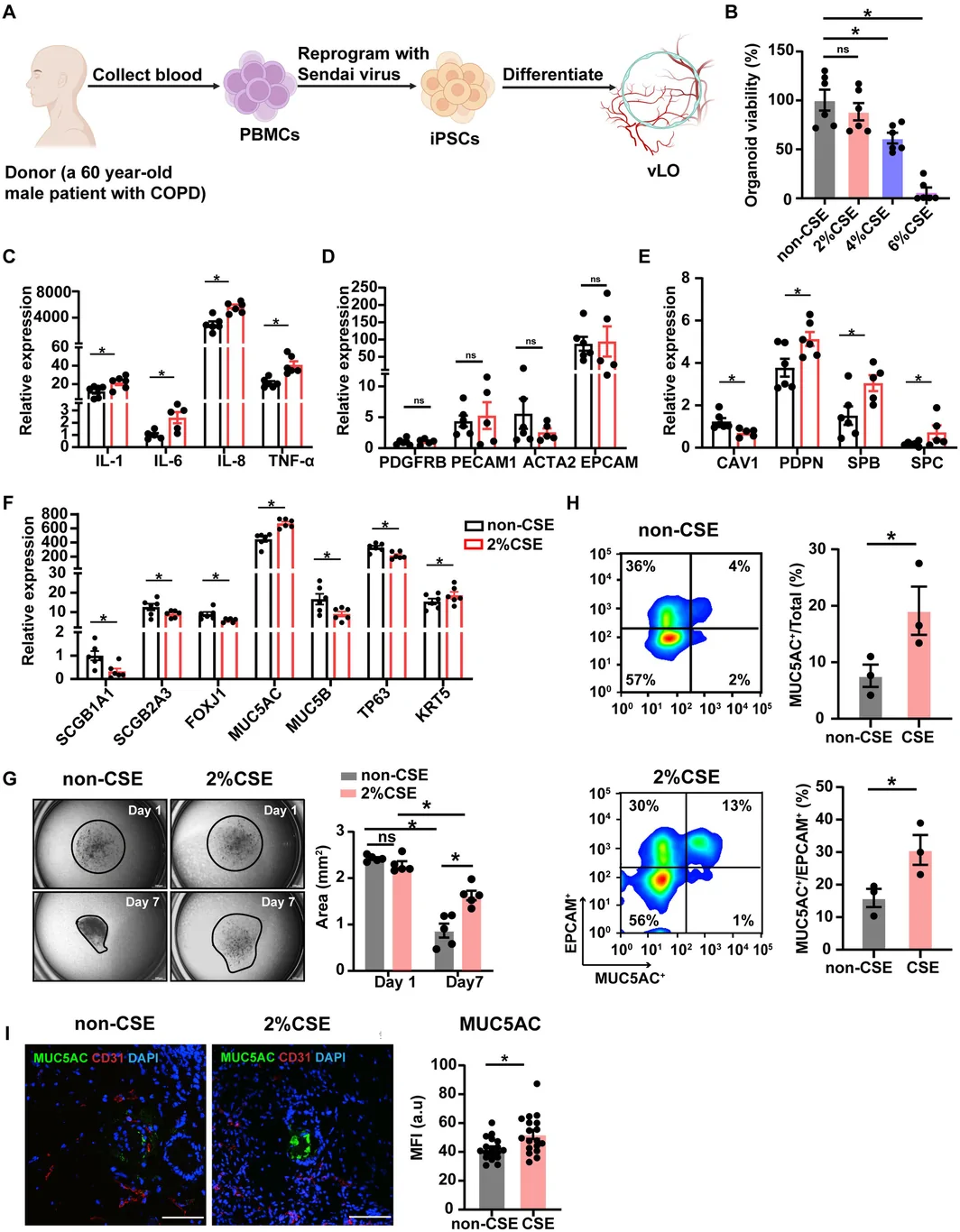
Cigarette smoke extract (CSE) treatment alters the cellular composition of vLOs. (A) Schematic illustrating the generation of vLOs from iPSCs derived from a COPD patient. (B) CCK8 assay measuring organoid viability after 2 weeks of treatment with different concentrations of CSE. *p < 0.05 vs. control, one‐way ANOVA followed by Dunnett's test. (C) RT‐qPCR analysis showing elevated cytokine expression induced by CSE treatment. Data are presented as mean ± SE. *p < 0.05, unpaired Student's *t*‐test. (D) RT‐qPCR results for relative expression of gene markers associated with fibroblasts (PDGFRB), endothelial cells (PECAM), smooth muscle cells (ACTA2) and epithelial cells (EPCAM) in CSE‐treated and untreated vLOs. Data are presented as mean ± SE. *p < 0.05, unpaired Student's *t*‐test. (E) RT‐qPCR results showing the relative expression of gene markers for AT1 (PDPN, CAV1) and AT2 (SPB, SPC). Data are presented as mean ± SE. *p < 0.05, unpaired Student's *t*‐test. (F) RT‐qPCR results showing the relative expression of gene markers for club cells (SCGB1A1, SCGB3A2), ciliated cells (FOXJ1), goblet cells (MUC5AC, MUC5B) and basal cells (TP63, KRT5). Data are presented as mean ± SE. *p < 0.05, unpaired Student's *t*‐test. (G) Whole‐well imaging and quantification of the area of cultivation matrix of vLOs on day 1 and day 7 in CSE‐treated group and the control group. Scale bars, 500 μm. Data are presented as mean ± SE. *n* = 5, *p < 0.05, unpaired Student's *t*‐test was used between CSE group and control. Paired Student's t‐test was used between day 1 and day 7. (H) Flow cytometry image and quantification of the proportion of goblet cells (MUC5AC^+^) among total cells or lung epithelial cells (CPM^+^) in vLOs. Data are presented as mean ± SE. *p < 0.05, unpaired Student's *t*‐test. (I) Immunofluorescence image of the vLOs and quantification of the mean fluorescence intensity of MUC5AC. Data are presented as mean ± SE (*n* = 6 independent biological replicates, with each replicate analysing three independent images). *p < 0.05, unpaired Student's *t*‐test.

With a mature, vascularised lung model in place, we next asked whether it could reproduce key features of human lung disease. COPD is a multifactorial condition in which cigarette smoke, air pollution and other environmental exposures are well‐established risk factors; however, growing evidence also highlights a significant genetic contribution to disease susceptibility [33]. To model COPD pathogenesis in a physiologically relevant context, we generated vLOs from iPSCs derived from a COPD patient with a 30‐pack‐year smoking history. Peripheral blood mononuclear cells were reprogrammed using Sendai virus‐mediated transduction and subsequently differentiated into vLOs (Figure 3A).

To reproduce the early events of disease initiation, we exposed these patient‐derived vLOs to subtoxic concentrations of CSE that approximated the patient's historical smoke exposure. Following 2 weeks of treatment with 2%–6% CSE, cell viability was assessed using CCK‐8 assays. The results indicated that 2% CSE did not significantly compromise organoid viability (Figure 3B) and was selected for further experiments.

Consistent with clinical COPD manifestations, vLOs treated with 2% CSE for 2 weeks showed significantly elevated expression of proinflammatory cytokines, including IL‐1β, IL‐6, IL‐8 and TNF‐α (Figure 3C). RT‐qPCR analysis of cellular markers revealed stable expression of mesenchymal (PDGFRB, ACTA2), endothelial (PECAM1) and general epithelial (EPCAM) markers (Figure 3D). However, we detected a marked upregulation of alveolar Type 1 (AT1; PDPN) and AT2 (SPB, SPC) markers, as well as goblet cell markers (MUC5AC), alongside a reduction in club cell (SCGB1A1, SCGB3A2) and ciliated cell (FOXJ1) markers (Figure 3E,F). Interestingly, while TP63 (basal progenitor marker) decreased, KRT5 (basal cytokeratin) significantly increased, consistent with epithelial metaplastic transition (Figure 3F).

Immunofluorescence and flow cytometry further confirmed goblet cell hyperplasia and increased MUC5AC secretion in CSE‐treated vLOs (Figure 3H,I). In addition to epithelial alterations, CSE exposure also markedly impaired vascular function. Specifically, we observed significantly reduced contractile capacity of vascular vessels, as demonstrated by reduced gel contraction in CSE‐treated vLOs compared to untreated controls (Figure 3G). Together, these results demonstrate that patient‐derived vLOs, when exposed to clinically relevant CSE doses, faithfully reproduce key structural, inflammatory and vascular dysfunction phenotypes characteristic of COPD.

### 4.4 Single‐Cell Profiling Reveals COPD‐Like Cellular and Molecular Alterations in CSE‐Treated vLOs

**FIGURE 4:**
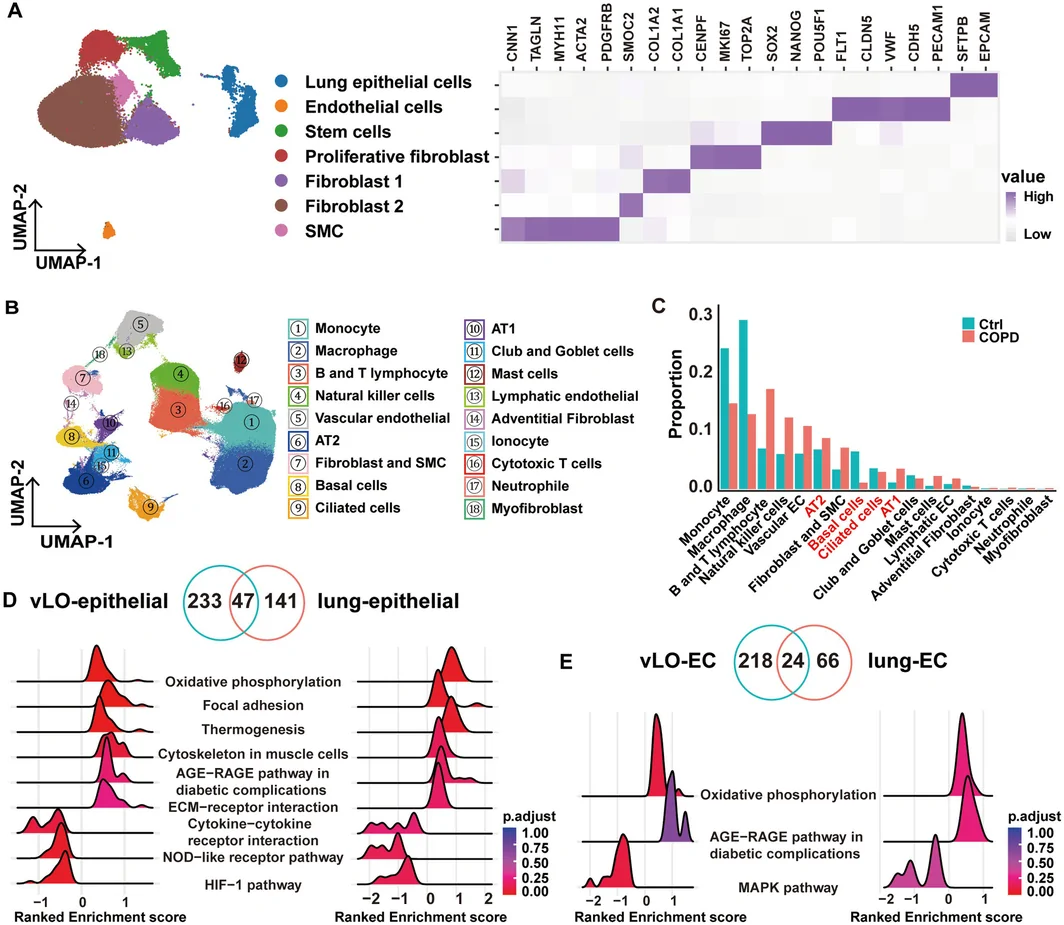
Single‐cell transcriptomic analysis of CSE‐treated versus non‐CSE vLOs and COPD vs. healthy lung tissues. (A) UMAP visualisation of single‐cell transcriptomic data from vLOs treated with CSE and non‐CSE controls. Left: UMAP visualisation of cellular heterogeneity. Right: Heatmap of cluster‐defining marker genes. (B) Batch‐corrected UMAP visualisation of major cell types in lung tissues from 51 COPD patients and 51 healthy controls. (C) Relative proportions of major cell types in COPD and healthy control groups. (D) KEGG pathway enrichment (GSEA) in epithelial cells. Left: Pathways enriched from DEGs (CSE vs. non‐CSE vLOs). Right: Pathways enriched from DEGs (COPD vs. healthy lungs). Venn diagram shows overlap. (E) KEGG pathway enrichment (GSEA) in endothelial cells. Left: Pathways enriched from DEGs (CSE vs. non‐CSE vLOs). Right: Pathways enriched from DEGs (COPD vs. healthy lungs). Venn diagram shows overlap.

To gain deeper insight into the cellular and molecular alterations induced by cigarette smoke exposure, we performed scRNA‐seq on both CSE‐treated and untreated organoids. UMAP visualisation identified distinct major cell populations, including lung epithelial cells, ECs, SMCs, fibroblasts and pluripotent stem cells (Figure 4A). Epithelial cell subclustering further resolved AT2 cells, ciliated cells, basal cells, goblet cells and a population of undefined epithelial progenitors (Figure S2A). Pseudotime trajectory analysis suggested a developmental continuum originating from basal cells and undefined progenitors, progressing towards differentiated lineages (ciliated cells, AT1, AT2 and goblet cells) (Figure S2B).

To validate the clinical relevance of our model, we integrated our data with scRNA‐seq datasets from 51 COPD patients and 51 healthy controls (GSE173896, GSE136831, GSE167295, GSE196638, GSE171541, GSE196341 and GSE168191) (Figure 4B) [17, 18, 19, 20, 21, 22]. Relative to healthy controls, the COPD group displayed an increased proportion of AT1 and AT2 cells, accompanied by a decreased proportion of basal and ciliated cells (Figure 4C). The proportion of secretory cells, which consist of goblet cells and club cells, was slightly reduced in the COPD group. Notably, the changes in cell proportions in CSE‐treated vLOs partially mirrored those observed in COPD patients, further validating the model's physiological relevance.

Gene set enrichment analysis (GSEA) identified 141 dysregulated pathways in COPD patient‐derived epithelial cells and 233 dysregulated pathways in CSE‐treated vLO epithelial cells (Figure 4D), with 47 pathways showing concordant alterations across both groups. These shared pathways included upregulated oxidative phosphorylation, focal adhesion, thermogenesis, muscle cell cytoskeleton organisation and the AGE‐RAGE signalling pathway in diabetic complications, while pathways related to cytokine‐cytokine receptor interaction, NOD‐like receptor signalling and HIF‐1 signalling were downregulated.

Independent GSEA analyses revealed 66 disrupted pathways in COPD patient epithelial cells and 218 in CSE‐treated vLO epithelial cells (Figure 4E), with 24 pathways demonstrating consistent alterations. Notably, both pulmonary endothelial cells and epithelial cells from COPD patients and from CSE‐treated vLOs showed upregulation of oxidative phosphorylation and AGE‐RAGE signalling pathways (Figure 4D,E), suggesting that these pathways may play a critical role in COPD pathogenesis and progression.

### 4.5 NaHS and Sotatercept Alleviates Endothelial‐To‐Mesenchymal Transition (EndMT) in vLOs Derived From PH Patient

**FIGURE 5:**
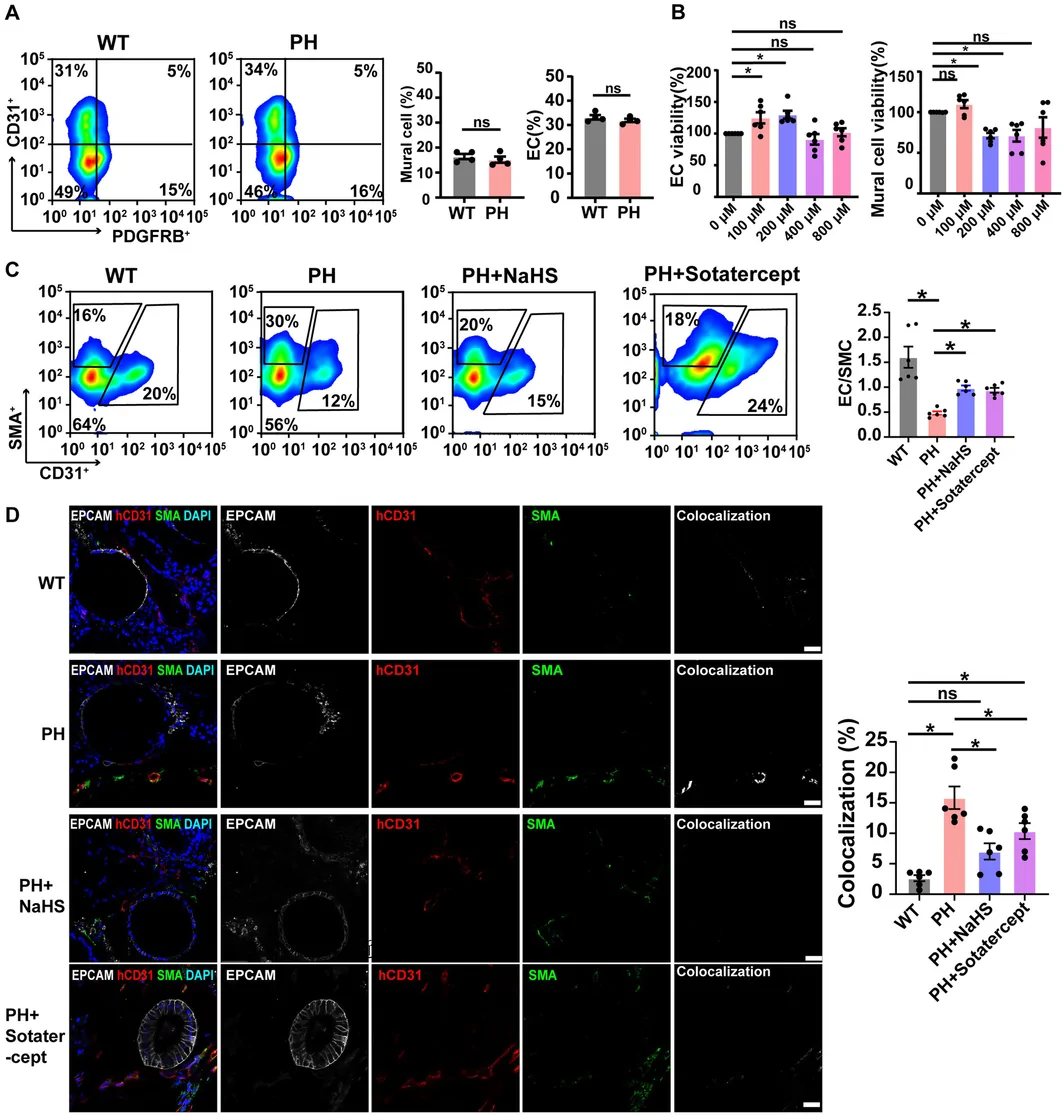
Therapeutic effect of NaHS on endothelial‐to‐mesenchymal transition (EndMT) in PH patient‐derived vLOs (A) Comparative analysis of iPSC differentiation efficiency between BMPR2‐mutant PH patient‐derived iPSCs and their isogenic BMPR2‐corrected counterparts (WT) by flow cytometry. Data represent mean ± SE (*n* = 3 biological replicates); *p < 0.05, unpaired Student's *t*‐test. (B) Dose‐dependent effect of NaHS on cell viability assessed by CCK‐8 assay in induced endothelial and mural cells. Control values (0 μM) were normalised to 100% within each independent biological replicates (*n* = 6) to account for inter‐experimental variability. Data represent mean ± SE; *p < 0.05 vs. control, one‐way ANOVA followed by Dunnett's test. (C) Dose‐dependent effect of sotatercept on cell viability assessed by CCK‐8 assay in induced endothelial and mural cells. Control values (0 μM) were normalised to 100% within each independent biological replicates (*n* = 6) to account for inter‐experimental variability. Data represent mean ± SE; *p < 0.05 vs. control, one‐way ANOVA followed by Dunnett's test. (D) Quantification of EC/SMC ratios through flow cytometry in three experimental groups: Isogenic control vLOs (WT), PH patient‐derived vLOs (PH), NaHS‐treated PH‐vLOs and sotatercept treated PH‐vLOs. Data represent mean ± SE (*n* = 6 biological replicates); *p < 0.05, One‐way ANOVA with Tukey's test. (E) Immunofluorescence characterisation of EndMT progression, featuring a composite image displaying hCD31^+^/SMA^+^ co‐localisation patterns across WT, PH, PH + NaHS and PH + sotatercept groups, along with channel decomposition (EPCAM, hCD31, SMA) and a co‐localisation view of hCD31 and SMA. Quantification of hCD31/SMA co‐localisation is shown (*n* = 6 biological replicates); *p < 0.05, One‐way ANOVA with Tukey's test. Scale bar: 100 μm.

To establish an in vitro model of PH, we generated vLOs using iPSCs derived from a PH patient harbouring a pathogenic *BMPR2* mutation (c.631 C>T). Isogenic control iPSCs were generated via CRISPR/Cas9‐mediated correction of the *BMPR2* mutation, as previously described [15]. Both mutant and corrected iPSC lines were differentiated into vascular organoids, with flow cytometric analysis confirming comparable differentiation efficiencies in generating ECs (CD31^+^) and mural cells (PDGFRβ^+^), which consist of SMC and pericyte, between groups (Figure 5A), indicating that the *BMPR2* mutation did not impair lineage specification.

Given the well‐documented cardioprotective effects of hydrogen sulfide (H_2_S) in vascular remodelling [34, 35]. we investigated the therapeutic potential of its donor compound, sodium hydrosulfide (NaHS). CCK‐8 assays on CD31^+^ ECs and PDGFRβ^+^ mural cells isolated from patient‐derived vascular organoids showed that 200 μM NaHS is the optimal dosage concentration, as it simultaneously enhanced EC proliferation and suppressed mural cell growth (Figure 5B), thereby potentially restoring the dysregulated EC/mural cell ratio characteristic of PH. To further validate the clinical relevance of PH‐vLOs, we administered sotatercept, a clinically approved PH therapy, to assess the organoid response. CCK‐8 assays revealed that both 10 ng/mL and 100 ng/mL sotatercept increased the viability of ECs and mural cells (Figure 5C); consequently, the lower concentration (10 ng/mL) was selected for subsequent experiments.

Following 2 weeks of vLOs formation, quantitative analysis revealed significant pathological alterations in PH‐derived vLOs: flow cytometry showed an abnormally decreased EC/SMC ratio compared to controls, which was rescued by NaHS and sotatercept treatment (Figure 5D). Importantly, immunofluorescence staining demonstrated pronounced CD31^+^/SMA^+^ co‐localisation in the ECs of PH‐vLOs, confirming enhanced EndMT. This pathological signature was substantially attenuated by NaHS and sotatercept administration (Figure 5E), supporting their efficacy in mitigating PH‐associated EndMT.

### 4.6 NaHS Alleviates Vascular Remodelling and Fibrosis in PH‐Patient‐Derived vLOs

**FIGURE 6:**
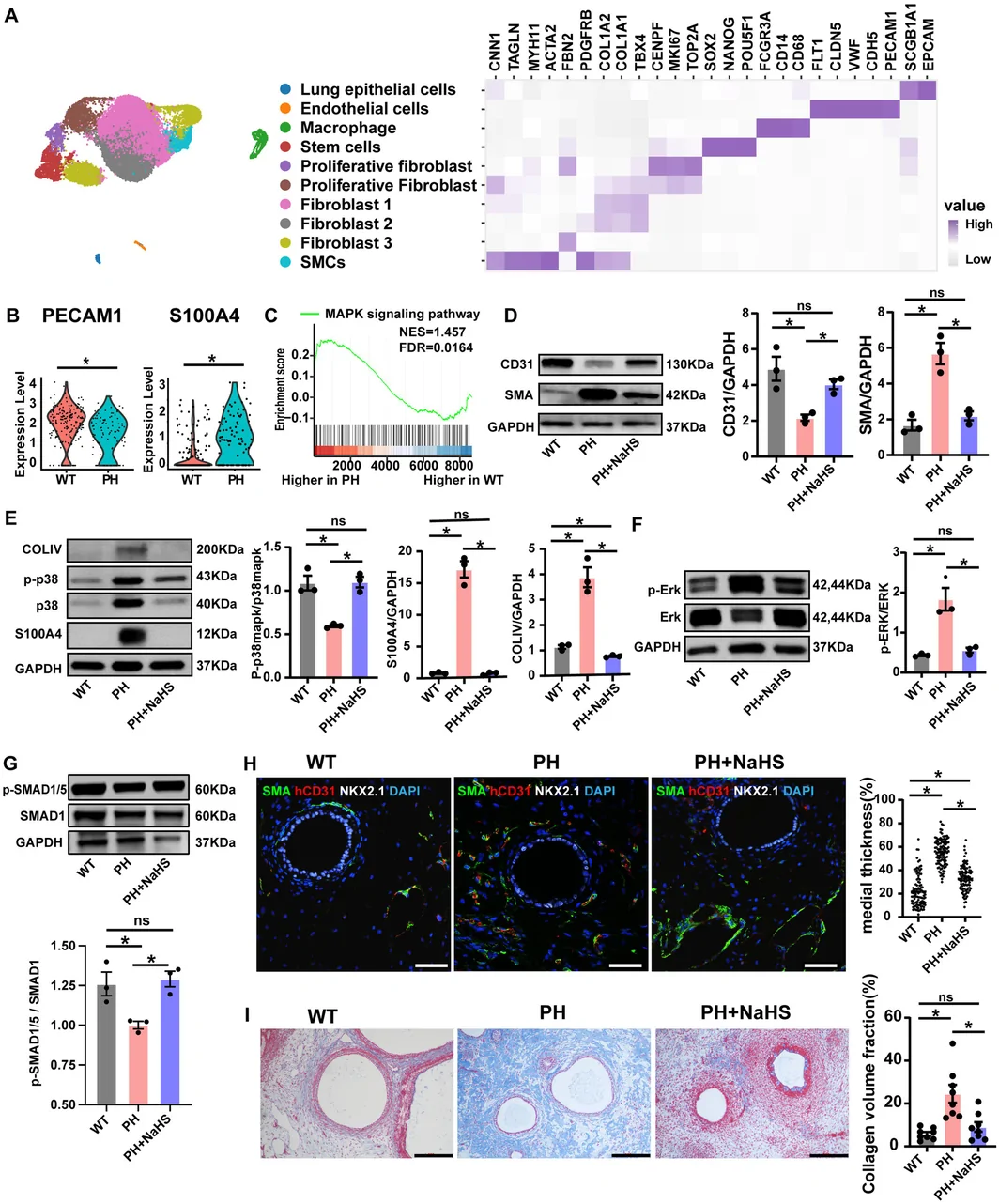
NaHS attenuates EndMT and promotes endothelial cell proliferation via the MAPK signalling pathway. (A) Single‐cell transcriptomic profiling of vLOs from PH patients and controls. Left: UMAP projection illustrating cellular heterogeneity. Right: Heatmap displaying cluster‐defining marker genes. (B) Violin plots showing differential expression of endothelial (PECAM1) and mesenchymal (S100A4) markers in ECs (*p < 0.05, One‐way ANOVA with Tukey's test). (C) Ridge plots of KEGG pathway enrichment analysis (GSEA) in endothelial cells. (D) Western blot quantification of endothelial (CD31/GAPDH) and mesenchymal (α‐SMA/GAPDH) markers (*mean ± SE, *n* = 3; *p < 0.05, One‐way ANOVA with Tukey's test). (E‐G) Western blot analysis of MAPK pathway activation (p‐p38/p38, p‐Erk/Erk), EndMT markers (Collagen IV, S100A4) and SMAD1/5 phosphorylation (pSMAD1/5/SMAD1) (*mean ± SE, *n* = 3; *p < 0.05, One‐way ANOVA with Tukey's test). (H) Representative immunofluorescence images of vascularised lung tissue and quantification of medial thickness in human vessels (*n = 8 replicates, three images each; *p* < 0.05, One‐way ANOVA with Tukey's test). Scale bar, 50 μm. (I) Masson's trichrome staining showing collagen deposition (blue) and Collagen volume fraction quantification (*n* = 8; *p < 0.05, One‐way ANOVA with Tukey's test). Scale bar, 200 μm.

Single‐cell sequencing of vLOs derived from PH patients and isogenic controls identified 6 major cell populations, including lung epithelial cells, ECs, SMCs, macrophages, fibroblasts and stem cells (Figure 6A). Analysis of ECs revealed decreased expression of the endothelial marker PECAM1 and increased expression of the mesenchymal marker S100A4 in PH‐patient‐derived vLOs compared to controls, suggesting EndMT (Figure 6B). GSEA of ECs further identified significant upregulation of MAPK signalling pathways (Figure 6C).

Consistent with the EndMT phenotype revealed by single‐cell sequencing, Western blot analysis showed that ECs derived from PH patients exhibited reduced CD31 expression and increased SMA expression relative to healthy controls. Treatment with 200 μM NaHS significantly inhibited SMA expression and restored CD31 levels in these PH‐derived ECs (Figure 6D). Similarly, Western blot analysis also validated elevated S100A4 and collagen IV expression in PH‐patient‐derived ECs, both of which were effectively reduced by NaHS treatment (Figure 6E). Furthermore, p38 MAPK expression and phosphorylation were markedly enhanced in PH ECs, reflecting pathway activation; NaHS treatment suppressed both p38 MAPK expression and phosphorylation (Figure 6E). Elevated Erk1/2 phosphorylation in the PH group was also attenuated by NaHS treatment (Figure 6F). These findings collectively suggest that NaHS inhibited EndMT, at least in part, by suppressing MAPK pathway activation. Furthermore, NaHS elevated the phosphorylation of SMAD1/5, which was decreased in the PH group (Figure 6G). In an in vivo setting, transplantation of PH‐patient‐derived vascularised lung tissue beneath the renal capsule of mice revealed a decreased internal‐to‐external media ratio of small arteries, indicating vascular remodelling (Figure 6H). Intraperitoneal administration of 25 μmol/kg/d NaHS for 3 weeks significantly increased this ratio. Additionally, NaHS alleviated fibrosis in the transplanted tissue, as evidenced by a significant reduction in collagen volume fraction (Figure 6I).

Taken together, these findings establish that NaHS therapy inhibits EndMT, ameliorates vascular remodelling and reduces fibrosis in PH‐patient‐derived models, highlighting its potential as a targeted therapeutic strategy for pulmonary hypertension.

## 5 Discussion

This study establishes iPSC‐derived vLOs as a physiologically relevant platform for modelling pulmonary diseases. The vLOs recapitulate human lung tissue morphology through an integrated vascular network and encompass a wide spectrum of lung cell types, including epithelial cells (AT1, AT2, basal, ciliated, goblet and club cells), endothelial cells (ECs), smooth muscle cells (SMCs), fibroblasts, macrophages and stem cells. Critically, the mesenchymal and endothelial characteristics within vLOs impart features suggestive of a lung‐like niche, thereby contributing to the modelling of parenchymal and mesenchymal pathologies.

vLOs derived from COPD patients accurately recapitulated cigarette smoke extract (CSE)‐induced epithelial remodelling—a well‐recognised hallmark of COPD [36, 37, 38]. Key features of COPD airway remodelling include goblet cell metaplasia [39, 40, 41], reduced club cell populations [40, 42, 43], squamous metaplasia [44, 45] and decreased ciliated cell numbers with dysfunction [46, 47]. RT‐qPCR analysis revealed that CSE‐treated vLOs exhibited significant upregulation of markers for AT1, AT2 and goblet cells, alongside significant downregulation of club and ciliated cell markers. Notably, CSE treatment induced divergent basal cell marker expression (TP63 downregulation, KRT5 upregulation), which potentially associates with squamous metaplasia [48]. Critically, these findings were validated using public single‐cell transcriptomics data from the NCBI database, which showed striking concordance with clinical COPD samples (51 patients vs. 51 controls): increased proportions of AT1/AT2 cells and reduced populations of basal and ciliated cells. GSEA revealed conserved dysregulation of oxidative phosphorylation and AGE‐RAGE signalling pathways in epithelial cells and ECs of both CSE‐exposed vLOs and clinical COPD specimens. Functionally, vLOs mirrored COPD phenotypes including impaired vascular contraction and mucous hypersecretion. These molecular and functional consistencies demonstrate that the vLO platform is an effective tool for modelling pathological epithelial and vascular alterations in COPD, providing a highly biomimetic research system for elucidating COPD pathogenesis and screening targeted therapeutics (e.g., agents targeting the AGE‐RAGE pathway).

BMPR2 mutations account for 70% of heritable PH [49]. Utilising vascularised lung organoids (vLOs) derived from BMPR2‐mutated patients, we established disease models that precisely recapitulate BMPR2 deficiency phenotypes, including impaired EC to SMC proportion in vitro and disrupted vascular network architecture in vivo. PH progression is characterised by vascular remodelling, where EndMT serves as a pivotal initiating event [50]. Significantly, we confirmed heightened EndMT in vLOs generated from BMPR2‐mutant PH patients versus healthy controls. Therapeutic intervention with NaHS—a hydrogen sulfide (H_2_S) donor that exerts vasoprotective effects—was found to inhibit the proliferation of induced mural cells while promoting the proliferation of induced ECs. NaHS administration attenuated EndMT in BMPR2‐mutant vLOs in vitro and mitigated vascular remodelling/pulmonary fibrosis in vivo in transplanted tissues. Collectively, BMPR2‐mutated PH‐patient‐derived vLOs effectively recapitulate PH pathophysiology and serve as a high‐fidelity platform for pharmacodynamic evaluation.

A current limitation of the vLO system is the absence of a fully integrated and functionally mature immune cell compartment. While macrophages are occasionally observed within the cultures, these cells arise as incidental byproducts of the differentiation process and do not represent functionally mature components of a stable immune system. Given the critical role of immune cells in pulmonary pathogenesis, our ongoing work focuses on stabilising macrophage generation and driving their functional maturation into pulmonary macrophages within vLOs, as well as incorporating other functionally competent immune cells, such as T cells, into this system. Another limitation is that the relative proportions of cell types in vLOs do not fully recapitulate in vivo tissue composition. Future optimisation of differentiation protocols and culture media may help achieve a more physiologically relevant cellular composition. Finally, because the current proof‐of‐concept model was derived from a single patient iPSC line, future studies employing multiple donor lines will be necessary to validate the disease‐specific findings.

## 6 Conclusion

In conclusion, this study establishes iPSC‐derived vLOs as an innovative physiomimetic platform for modelling pulmonary diseases. Notably, these vLOs recapitulate the complex pathology of COPD, encompassing epithelial remodelling, vascular dysfunction and dysregulated signalling; model the EndMT‐driven mechanisms underlying vascular remodelling in PH; and enable therapeutic validation, as exemplified by NaHS‐mediated inhibition of EndMT with concomitant attenuation of vascular remodelling and fibrosis, both in vitro and in vivo. vLOs thus represent a transformative human‐relevant system with significant potential for mechanistic discovery and drug screening in COPD, PH and related pulmonary disorders.

## Author Contributions

S.J., L.T. and J.Q. contributed equally to this work. S.J., K.W. and Y.C. conceived and designed experiments. L.T. and Z.P. designed the gene‐editing cell lines. L.T. and J.Q. performed the bioinformatic analysis. S.J., L.T., J.Q., Z.P., D.C., Y.H., H.L., L.C., X.M., Y.Z., B.M., K.W. and Y.C. wrote and reviewed the manuscript. All authors read and approved the manuscript.

## Funding

This work was supported by the National Natural Science Foundation of China (82090014, 82370514, 92468105, 82522010, U24A20375, 82570058), the National Key Research and Development Program of China (2022YFC3401100), the Natural Science Foundation of Beijing (J230030, JQ23029), Open Project of the State Key Laboratory of Vascular Homeostasis and Remodeling (2025‐VHR‐O‐SY‐01), and National Natural Science Foundation of China Cooperation and Exchange Program (W2421096).

## Conflicts of Interest

The authors declare no conflicts of interest.

## Supporting information


**Figure S1:** Generation and characterisation of vLOs.
(A) Protocol for vLO generation through coculture of airway, alveolar and vascular organoids. Representative bright‐field images show cellular morphology and organoid formation at each developmental stage. Scale bar: 100 μm (B–D) Immunophenotypic profiling of vLO components: (B) Endothelial network (CD31^+^) alongside basal cells (KRT5^+^). (C) Vascular structures (CD31^+^) adjacent to club cells (SCGB3A2^+^). (D) EC (CD31^+^), SMC (SMA^+^) and lung epithelial cells (EPCAM^+^) in the vLOs and collage IV (COL IV^+^) was exhibited along the endothelial cells and epithelial cells. (E) Lung‐specific EC signatures. Dot plot showing expression of: bronchial EC markers (PLVAP, HSPG2, HBEGF), general capillary markers (gCap; PTPRB, EDN1) and alveolar capillary markers (aCap; S100A4, EMCN). colour intensity shows mean expression. (F) Dot plot showing expression of Lung‐specific mesenchymal signatures: TBX3, TBX4, TBX5.

**Figure S2:** Recluster of lung epithelial cells in vLOs.
(A) UMAP visualisation of re‐clustered epithelial cells from CSE‐treated and non‐CSE control vLOs. (B) Pseudotime trajectory analysis reveals cell state transitions of lung epithelial cells in vLOs.

**Movie S1:** 3D immunofluorescence image of vascularised lung organoids.

**Table S1:** Primer List.

**Table S2:** Reagent List.

## Data Availability

The data that support the findings of this study are openly available in CNCB at https://www.cncb.ac.cn/, reference number PRJCA034912.
