# Supplementary material for: Human iPSC‐Derived Vascularised Lung Organoids for Modelling COPD and Pulmonary Hypertension
Source: Cell Prolif. 2026 Sep 1:e70273. Online ahead of print. doi: 10.1111/cpr.70273 (PMC13531628; doi:10.1111/cpr.70273)
Supplement: Supplementary file 1 [file CPR-9999-e70273-s002.docx]

**Table 2. Reagent List**

| **Reagent** | **Company** | **CAT** |
| --- | --- | --- |
| **Antibody** |  |  |
| APC anti-CD31 | BioLegend | 303116 |
| Mouse anti-MUC5AC | Thermo Fisher Scientific | MA5-12178 |
| Mouse anti-CPM | Novus | DDX0520P |
| Mouse anti-ACT | Santa Cruz Biotechnology | sc-8432 |
| Mouse anti-α-SMA | Santa Cruz | sc-32251 |
| Mouse anti-GAPDH | ZSGB-Bio | TA-08 |
| Mouse anti-CD68 | Thermo Fisher Scientific | 14-0688-82 |
| PE-CD31 | BioLegend | 303106 |
| PE anti-PDGFRβ | Biosciences | 558821BD |
| Rat anti-CD326 (EpCAM) | Biolegend | 624152 |
| Rabbit anti-SFTPB | abcam | Ab271345 |
| Rabbit anti-SCGB3A2 | Abcam | ab181853 |
| Rat anti-SCGB1A1 | [R&D Systems](https://www.rndsystems.com/) | MAB4218 |
| Rabbit anti-KRT5 | cell signaling | 71536S |
| Rabbit anti-NKX2.1 | abcam | ab76013 |
| Rabbit anti p38 MAPK | cell signaling | 9212 |
| [Rabbit anti Phospho-p38 MAPK](https://www.cellsignal.cn/products/primary-antibodies/phospho-p38-mapk-thr180-tyr182-d3f9-xp-rabbit-mab/4511) | cell signaling | 4511 |
| Rabbit anti S100A4 | cell signaling | 13018 |
| Rabbit anti Collagen IV | abcam | Ab214417 |
| [Rabbit anti p44/42 MAPK (Erk1/2)](https://www.cellsignal.cn/products/primary-antibodies/p44-42-mapk-erk1-2-antibody/9102) | cell signaling | 9012 |
| Rabbit anti [Phospho-p44/42 MAPK (Erk1/2)](https://www.cellsignal.cn/products/primary-antibodies/phospho-p44-42-mapk-erk1-2-thr202-tyr204-197g2-rabbit-mab/4377) | cell signaling | 4377 |
| Rabbit anti Phospho-SMAD1/5 | cell signaling | 9516 |
| Rabbit anti SMAD1 | Abclonal | A19113 |
| Rabbit anti-KU80 | cell signaling | 2180 |
| Sheep anti-CD31 | R&D systems | AF806 |
| Syrian hamster anti-PDPN | Thermo Fisher Scientific | 14-5381-82 |
| Donkey anti-rabbit IgG, Alexa Fluor 568 | Thermo Fisher Scientific | A10042 |
| Donkey anti-rabbit IgG, Alexa Fluor 647 | Thermo Fisher Scientific | A-31573 |
| Donkey anti-goat IgG, Alexa Fluor 488 | Thermo Fisher Scientific | A-11055 |
| Donkey anti-goat IgG, Alexa Fluor 594 | Thermo Fisher Scientific | A-11058 |
| Donkey anti- goat IgG, Alexa Fluor 647 | Thermo Fisher Scientific | A-21447 |
| Donkey anti- mouse IgG, DyLight 755 | Thermo Fisher Scientific | SA5-10171 |
| Donkey anti- rabbit IgG, DyLight 755 | Thermo Fisher Scientific | SA5-10035 |
| Goat anti-mouse second antibody, HRP | Thermo Fisher Scientific | A16066 |
| Goat anti-rabbit second antibody, HRP | Thermo Fisher Scientific | 65-6120 |
| **Chemicals, peptides, and recombinant proteins** |  |  |
| Anti-R-Phycoerythrin (PE) Magnetic Particle-DM | BD Biosciences | 557899 |
| Activin A |  |  |
| AceQ qPCR SYBR Green Master Mix | Vazyme | Q121 |
| Bovine serum albumin (BSA), Fatty acid free | Proliant | 68700 |
| B-27 | Yeasen | 60703ES10 |
| BCA kit | Beyotime | P0012 |
| 2-Mercaptoethanol (BME) | Sigma | M3148 |
| 8-Bromo-cAMP | TargetMol | T6747 |
| Advanced DEME/F-12 | Thermo Fisher Scientific | 12634028 |
| Collagenase I | Gibco | 17101015 |
| Collagenase II | Gibco | 17100017 |
| Cell Recovery Solution | Corning Biocoat | 354253 |
| CHIR99021 | Topscience | T2310 |
| Cell Counting Kit (CCK-8) | Yeason | 40203ES76 |
| [CytoVista Tissue and 3D Culture Clearing for 3D Fluorescence Imaging](https://assets.thermofisher.cn/TFS-Assets/LSG/manuals/MAN0017942_CytoVista_Tissue_and_3D_Cell_Culture_Clearing_UG.pdf) | Thermo Fisher Scientific | v11326 |
| DAPT | TargetMol | T6202 |
| Dexamethasone | TargetMol | T1076 |
| DMEM/F12 | Gibco | C11330500BT |
| DAPI solution | Solarbio | C0060 |
| Dispase | Stemcell | 07923 |
| Dorsomorphin (DSM) | TargetMol | T6146 |
| EDTA Antigen Retrieval Solution (pH 8.0) | Solarbio | C1033 |
| ECL Reagent | Beyotime | P0018S |
| Fluoroshield Mounting Medium With DAPI | Abcam | ab104139 |
| Fetal Bovine Serum (FBS) | Thermo Fisher Scientific | 10099158 |
| FACS Stain Buffer | BD Biosciences | 554657 |
| 10% Formalin | Sigma-Aldrich | F5554 |
| Glutamax | Thermo Fisher Scientific | 35050061 |
| HiScript III RT SuperMix Kit | Vazyme | R323-01 |
| IBMX | TargetMol | T1713 |
| L690,330 | R&D systems | 0681 |
| L-ascorbic acid | Sigma-Aldrich | A8960 |
| mTesR1 medium | Stemcell Technologies | 85850 |
| MCDB131 | Sigma-Aldrich | [M8537](https://www.sigmaaldrich.cn/CN/zh/product/sigma/m8537) |
| Matrigel Growth Factor Reduced (GFR) Basement Membrane Matrix | Corning | 354230 |
| Matrigel hESC-Qualified Matrix | Corning | 354277 |
| N-2 | Yeasen | 60706ES08 |
| Penicillin/streptomycin | Thermo Fisher Scientific | 15-140-122 |
| PBS, pH7.4 | Corning | 21-040-CV |
| PureCol(R) EZ Gel solution | Sigma-Aldrich | 5074 |
| Penicillin-streptomycin | Procell system | PB180120 |
| Recombinant human ActivinA | Sino Biological | 10429-HNAH |
| Recombinant human EGF | Sino Biological | 10605-HNAE |
| Recombinant human VEGFA | Sino Biological | 11066-HNAH |
| Recombinant human FGF2 | Sino Biological | 10014-HNAE |
| Recombinant human FGF10 | Sino Biological | 10573-HNAE |
| Recombinant human BMP4 | Novoprotein | CR93 |
| Recombinant human KGF/FGF-7 | Sino Biological | 10210-H07E |
| Resveratrol (RESV) | Sigma-Aldrich | R5010 |
| Retinoic acid | Sigma-Aldrich | R2625 |
| SB431542 | Topscience | T1726 |
| Sotatercept | MedChemExpress | HY-P99590 |
| TrypLE Express Enzyme (1X) | Thermo Fisher Scientific | 12604013 |
| Trypsin-EDTA, 0.05% | Thermo Fisher Scientific | 25300054 |
| Tween-20 | Solarbio | T8220 |
| Triton X-100 | Sigma-Aldrich | X100-500ML |
| TransZol Up Plus RNA KitTransZol Up Plus RNA Kit | Transgene | ER501-01-V2 |
| Y-27632 | TargetMol | T1725 |
