# Supplementary material for: Human iPSC‐Derived Vascularised Lung Organoids for Modelling COPD and Pulmonary Hypertension
Source: Cell Prolif. 2026 Sep 1:e70273. Online ahead of print. doi: 10.1111/cpr.70273 (PMC13531628; doi:10.1111/cpr.70273)
Supplement: Supplementary file 2 [file CPR-9999-e70273-s003.docx]

**Key resources table**

**Table 1. Primer List**

| **Primer List** | **Forward sequence (5'to3')** | **Reverse sequence (5'to3')** |
| --- | --- | --- |
| GAPDH | GGTGTGAACCATGAGAAGTATGA | GAGTCCTTCCACGATACCAAAG |
| PDGFRb | TGCAGACATCGAGTCCTCCAAC | GCTTAGCACTGGAGACTCGTTG |
| EPCAM | AGAACCTACTGGATCATCATTGAACTAA | CGCGTTGTGATCTCCTTCTG |
| ACTA2 | CTATGCCTCTGGACGCACAACT | CAGATCCAGACGCATGATGGCA |
| CD31 | AAGTGGAGTCCAGCCGCATATC | ATGGAGCAGGACAGGTTCAGTC |
| SCGB3A2 | GGCTAAGGAAGTGTGTAAATGAGC | CCATCCACCTCCGCTCTTTATC |
| SCGB1A1 | CACCATGAAACTCGCTGTCAC | AGTTCCATGGCAGCCTCATAAC |
| SPC | CACCTGAAACGCCTTCTTATCG | TTTCTGGCTCATGTGGAGACC |
| SPB | TGTCCTCCGATGTTCCACTGAG | AGCCTGTTCACTGGTGTTCCAG |
| MUC5AC | CATCTGCCAGCTGATTCTGA | AAGACGCAGCCCTCATAGAA |
| MUC5B | CTGCTACGACAAGGACGGAAAC | AAGGCTGTGAGCGCACTGGATG |
| FOXJ1 | CCTGTCGGCCATCTACAAGT | AGACAGGTTGTGGCGGATT |
| PDPN | TCCAGGAACCAGCGAAGAC | CGTGGACTGTGCTTTCTGA |
| CAV1 | CCAAGGAGATCGACCTGGTCAA | GCCGTCAAAACTGTGTGTCCCT |
| TP63 | ACTGCCAAATTGCAAAGACA | TGACTAGGAGGGGCAATCTG |
| KRT5 | GAGCTGAGAAACATGCAGGA | TCTCAGCAGTGGTACGCTTG |
| IL6 | ACTCACCTCTTCAGAACGAATTG | CCATCTTTGGAAGGTTCAGGTTG |
| IL8 | ACTGAGAGTGATTGAGAGTGGAC | AACCCTCTGCACCCAGTTTTC |
| TNF | CCTCTCTCTAATCAGCCCTCTG | GAGGACCTGGGAGTAGATGAG |
| IL1β | ATGATGGCTTATTACAGTGGCAA | GTCGGAGATTCGTAGCTGGA |
